# Microbial Systems Ecology to Understand Cross-Feeding in Microbiomes

**DOI:** 10.3389/fmicb.2021.780469

**Published:** 2021-12-20

**Authors:** Alice Mataigne, Nathan Vannier, Philippe Vandenkoornhuyse, Stéphane Hacquard

**Affiliations:** ^1^Université de Rennes 1, CNRS, UMR6553 ECOBIO, Rennes, France; ^2^Max Planck Institute for Plant Breeding Research, Cologne, Germany

**Keywords:** cross-feeding, microbiota, system ecology, metabolic interaction, coexistence

## Abstract

Understanding how microorganism-microorganism interactions shape microbial assemblages is a key to deciphering the evolution of dependencies and co-existence in complex microbiomes. Metabolic dependencies in cross-feeding exist in microbial communities and can at least partially determine microbial community composition. To parry the complexity and experimental limitations caused by the large number of possible interactions, new concepts from systems biology aim to decipher how the components of a system interact with each other. The idea that cross-feeding does impact microbiome assemblages has developed both theoretically and empirically, following a systems biology framework applied to microbial communities, formalized as microbial systems ecology (MSE) and relying on integrated-omics data. This framework merges cellular and community scales and offers new avenues to untangle microbial coexistence primarily by metabolic modeling, one of the main approaches used for mechanistic studies. In this mini-review, we first give a concise explanation of microbial cross-feeding. We then discuss how MSE can enable progress in microbial research. Finally, we provide an overview of a MSE framework mostly based on genome-scale metabolic-network reconstruction that combines top-down and bottom-up approaches to assess the molecular mechanisms of deterministic processes of microbial community assembly that is particularly suitable for use in synthetic biology and microbiome engineering.

## Introduction

**Figure 1:**
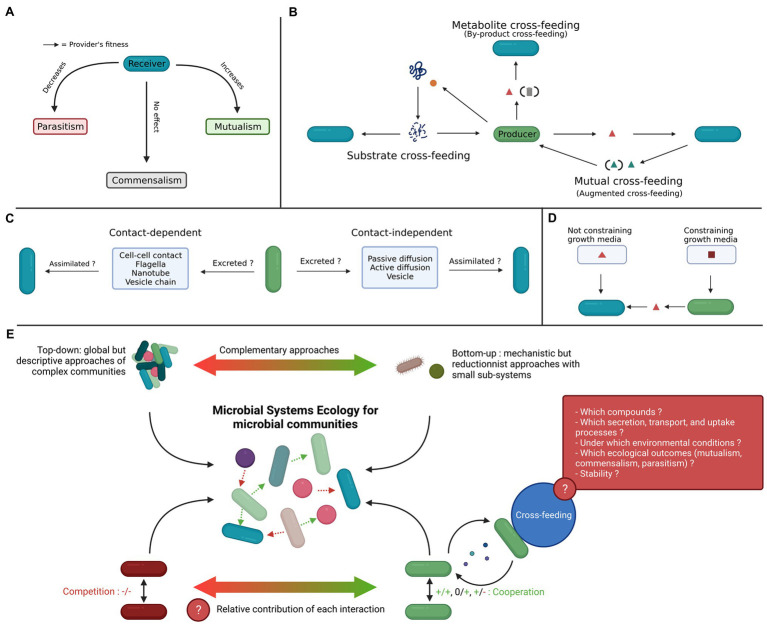
Cross-feeding among co-occurring microorganisms and its integration to microbial systems ecology (MSE). **(A)** Symbiosis is the interaction between living entities along a gradient from mutualism to parasitism, depending on the effect of the receiver (also referred to as “beneficiary,” blue bacteria symbols) on the fitness of the provider (green bacteria symbols). **(B)** There are several subcategories of cross-feeding (Smith et al., 2019). The type of secreted compounds, i.e., wastes (garbage icons) or other metabolites (red triangles) and on the directionality of the exchange (mutual or not, blue triangles) particularly matter in the classification of cross-feeding (see glossary for associated definitions). Enzymes (orange circle) can also be secreted to degrade complex molecules, making them available both for the producer and the receiver(s). **(C)** The existence of cross-feeding depends on the secretion, transport, and assimilation capacity of the public good (D’Souza et al., 2018). **(D)** Metabolic interactions are environment-dependent, notably regarding available nutrients. If a required nutrient (red triangle) is freely available in the growth medium, then cross-feeding is not indispensable for the receiver organism. Otherwise, when a particular nutrient is not available, but is synthesized by the producer from another substrate (brown square), cross-feeding becomes obligatory for the receiver. **(E)** Graphical abstract summarizing the study of ecological interactions, notably cross-feeding, in microbial communities with MSE.

Deciphering the assembly rules of microbial communities is vital for a mechanistic understanding of the general principles driving microbiome activity and functions (Vellend et al., 2014; Morrison-Whittle and Goddard, 2015). Microbial communities are governed by both stochastic and deterministic factors (Vellend, 2010; Stegen et al., 2012), and recent advances show that deterministic processes largely contribute to shaping microbial community assembly. Their relative contribution varies however according to the ecology of microorganisms (e.g., specialists or generalists) and the stability of the environment (Figure 1E, Stegen et al., 2012; Ning et al., 2020; Xu et al., 2020). Ecological interactions including commensalism, competition, and mutualism contribute to the self-organizational properties of microbiomes (Stegen et al., 2013). However, how these different interactions act in concert to shape microbial assemblages remain poorly understood (Nemergut et al., 2013). Microbial communities are likely not only driven by antagonistic interactions but also by cooperative symbioses, defined in 1879 by De Bary (2019) as the “living together of unlike organisms.” Symbioses (thus cooperation) are now recognized as central drivers of (co-)evolution, and are often associated with obligate mutualism but are actually a continuum of interactions between mutualism and parasitism (Ewald, 1987; Drew et al., 2021), implying dependency of one organism on another (Figure 1A; Raina et al., 2018). Among these interactions, metabolic dependencies by cross-feeding likely explain patterns in microbial communities (Mas et al., 2016; Zomorrodi and Segrè, 2017; Amor and Bello, 2019; Coyte and Rakoff-Nahoum, 2019; Pacheco and Segrè, 2019; Seif et al., 2020; Zhu et al., 2020). In community ecology, competition and related competitive exclusion were previously considered to be the main drivers of community assembly. The competitive exclusion principle (also often referred to as Gause’s law) states that two species with the same ecological niche cannot coexist because of competition, which leads either to the extinction of species or to the differentiation of their ecological niche (Gause, 1960; Hardin, 1960; Pocheville, 2015). This role of competition was questioned by the observation of unexpectedly complex microbial communities according to general ecology theories (Pacheco and Segrè, 2019). Hence, cross-feeding is increasingly believed to play an important role in the complexity of microbial communities (Zengler and Zaramela, 2018). In this mini-review, we summarize the definitions of cross-feeding and its underlying mechanisms, as well as its importance in structuring microbial communities. Then, we describe microbial systems ecology (MSE), a discipline at the crossroads of systems biology and microbial community ecology aiming to explain coexistence.

## Metabolic Cross-Feeding as a Major Driver of Microbiota Assemblages

### Definitions and Examples of Cross-Feeding

Microbial cross-feeding (Figure 1) refers to the interaction between microorganisms in which molecules resulting from the metabolism of one microorganism (referred to as the provider or producer) are further metabolized by another (referred to as the receiver, or beneficiary, Figure 1B and glossary, Smith et al., 2019). Currently, microbial cross-feeding has been computationally predicted to be frequent in microbial communities. However, only experimental validation allowed to identify cases of cross-feeding (see examples for wild and engineered microorganisms in Mee et al., 2014)and (Shou et al., 2007). Thus, even if cross-feeding is likely frequent in nature, some aspects are still unclear. Notably, the species benefiting from cross-feeding and the compounds involved are not systematically known (but the diversity of known cases suggests there are not limited to a few compounds or species). Interestingly, specific environmental constraints such as nutrient limitation have been identified to favor cross-feeding. Microbial cross-feeding might not be limited to pairs of interacting microorganisms, as several receiver species could benefit from the metabolites of the same provider species. Cross-feeding can be either optional or obligatory for the survival of the microorganisms (Zengler and Zaramela, 2018). Different types of cross-feeding are recognized depending on whether they are unidirectional (one microorganism benefits from another) or bidirectional (both microorganisms benefit from each other’s secretions) or depending on which compounds are exchanged (Figure 1B and glossary, D’Souza et al., 2018; Smith et al., 2019). Similarly, cross-feeding has different ecological outcomes depending on the directionality. Shortly, unidirectional cross-feeding is equivalent to commensalism and bidirectional cross-feeding can be considered as mutualism. However, regarding the many different types of cross-feeding (Figure 1B), this statement is an oversimplification (see D’Souza et al., 2018; Smith et al., 2019; for a classification of cross-feeding). A closely associated term to cross-feeding is syntrophy, which also defines the consumption of an organism’s secretion by an auxotrophic organism (Smith et al., 2019). The definition however varies from obligatory to optional mutualistic metabolism (Morris et al., 2013; Hillesland, 2018). For example, sulfate-reducing bacteria are able to uptake sulfate both from sediments and from secretion of methanogenic bacteria, highlighting the advantage of optional cross-feeding flexibility (Plugge et al., 2011). The compounds involved also vary, and are sometimes restricted to waste products (Oliveira et al., 2014), sometimes not (Stams and Plugge, 2009; Pande and Kost, 2017).

One example of known mutual cross-feeding is between *Rhodococus ruber* and *Bacillus cereus*. *Rhodococus ruber* degrades a tetrahydrofuran, which results in acidic metabolites that are utilized by *B. cereus*, which, in return, regulates pH and secretes micronutrients that are essential for *R. ruber* (Liu et al., 2019). Less specific cross-feeding can also occur. For instance, *Akkermansia muciniphila* degrades and ferments its host’s mucus, leading to the production of oligosaccharides that are available for other microorganisms (Belzer et al., 2017). A hierarchy in the importance of microorganisms for the microbiota stability has also been demonstrated in relation to cross-feeding, using a species-deletion approach in a consortium of 14 bacteria (Gutiérrez and Garrido, 2019). In this study, the exclusion of most species did not affect the global growth of the community, except for *Bacteroides dorei*, whose deletion affected negatively 10 other species. *Bacteroides dorei* was required for lactate availability, a common good for the microbial consortium, making *B. dorei* a keystone species. Cross-feeding can also enable degradation of complex molecule chains, such as chitin. Various microorganisms are known to grow on chitin without known chitinase activity, pointing to cross-feeding cascades, from chitin degrading microorganisms to other microorganisms benefiting from degradation products (Beier and Bertilsson, 2013; Raimundo et al., 2021).

### Mechanisms Behind Cross-Feeding

One key process is extracellular secretion of a wide range of “public goods,” including enzymes, proteins, byproducts, waste, co-factors, amino-acids, and vitamins. They benefit all the organisms in the community that are able to assimilate them (Croft et al., 2005; Yu et al., 2009; Seth and Taga, 2014; Rodionova et al., 2015; Cavaliere et al., 2017; Zengler and Zaramela, 2018; Fritts et al., 2021). Many microorganisms are auxotrophic for various metabolites, lack essential pathways or genes, and thus rely on extracellular sources (Mee et al., 2014), which can thus be obtained by the secretions of other organisms.

However, a microorganism predicted to produce a compound does not necessarily secrete it. In addition, if secreted, the compound may have to be transported through the environment, and the other microorganisms have to be able to uptake it (Figure 1C, Sung et al., 2017; D’Souza et al., 2018; Zengler and Zaramela, 2018). Moreover, ecological interactions are affected by temporal and spatial patterns (Kelsic et al., 2015), and by the organisms’ surrounding environment (Bakker et al., 2014). Notably, available nutrients control the metabolic activity of microorganisms, whether or not they depend on others (Figure 1D, Heinken and Thiele, 2015; Magnúsdóttir et al., 2017). For example, when nutrients are limited, microorganisms can compensate by engaging in behaviors that facilitate nutrient acquisition, notably by excreting molecules that promote cross-feeding (Fritts et al., 2021), even if they usually compete (Zengler and Zaramela, 2018). Another example of an environmental effect involves two mutants of *Pseudomonas stuzeri*. Depending on the pH, the mutants can shift from competition to strong cross-feeding of nitrite, which is a toxic compound at low pH (Borer et al., 2020).

Gene loss is a major cause of auxotrophy, which may arise when a costly function can be performed by one or more members of the community (Boon et al., 2014; D’Souza et al., 2014; Mas et al., 2016; Meijer et al., 2020). Energy saving and fitness gain could account for the origin of frequent occurrences of auxotrophy in microorganisms as an evolutionary trajectory to escape competition toward a steady-state equilibrium for the coexistence of microorganisms (Mas et al., 2016). First, the cost of producing certain metabolites is avoided by obtaining them from the environment (Zengler and Zaramela, 2018). Second, mutual cross-feeding has been shown to reduce the energetic cost of some metabolic pathways, for example amino-acids biosynthesis (Mee et al., 2014). Metabolic exchanges thus divide the cost of labor (Thommes et al., 2019). However, predictions indicate that costless secretions may be numerous and represent sources of cross-feeding opportunities (Pacheco et al., 2019). Nevertheless, the evolution and stability in time of cooperative behaviors are not fully understood, because of the constant threat of the emergence of cheaters that benefit from the cooperative interactions but do not contribute to them (Cavaliere et al., 2017). Several studies explored and partially resolved this issue, but are beyond the scope of this paper. For detailed examples of frameworks, we recommend studies using evolutionary game theory (see glossary, Gore et al., 2009; Zomorrodi and Segrè, 2017) and the Black Queen Hypothesis (see glossary, Morris et al., 2012; Morris, 2015; Mas et al., 2016).

### The Growing Importance of Metabolic Cross-Feeding Compared to Competition

Previous work suggested that microbiota are dominated by competition (Foster and Bell, 2012; Venturelli et al., 2018; Coyte and Rakoff-Nahoum, 2019). However, results vary and although some studies suggest that microbial communities are governed by antagonistic interactions and rarely cross-feed or cooperate (Biggs et al., 2017; Venturelli et al., 2018), others revealed rich networks of metabolic interactions among microorganisms (Medlock et al., 2018). However, only in a few cases has interspecies cooperation been validated so far (Coyte and Rakoff-Nahoum, 2019). Nevertheless, niche differentiation and metabolic dissimilarity between co-occurring microorganisms could be explained by complementary biosynthetic capabilities thus microbial facilitation rather than by competitive exclusion (Zelezniak et al., 2015). The fact that several bacterial taxa cannot be grown alone *in vitro* could result from such dependencies (Mas et al., 2016). Recently, genome-scale metabolic modeling across thousands of habitats found that microbial communities spread along a competitive-cooperative axis, the most competitive microorganisms were characterized by larger genomes and were mainly present in soil, while the most cooperative ones had smaller genomes and were present in both free-living and host-associated habitats (Machado et al., 2021). Many communities seemed to be engaged in a trade-off between competition and cooperation, echoing the trade-off faced by microorganisms about being independent and depending on surrounding microorganisms (Thommes et al., 2019). Hence, in order to explain why microbial communities display so many species, coexistence in microbiomes is now investigated under frameworks that differ and complement the usual competitive exclusion principle.

**Figure 2:**
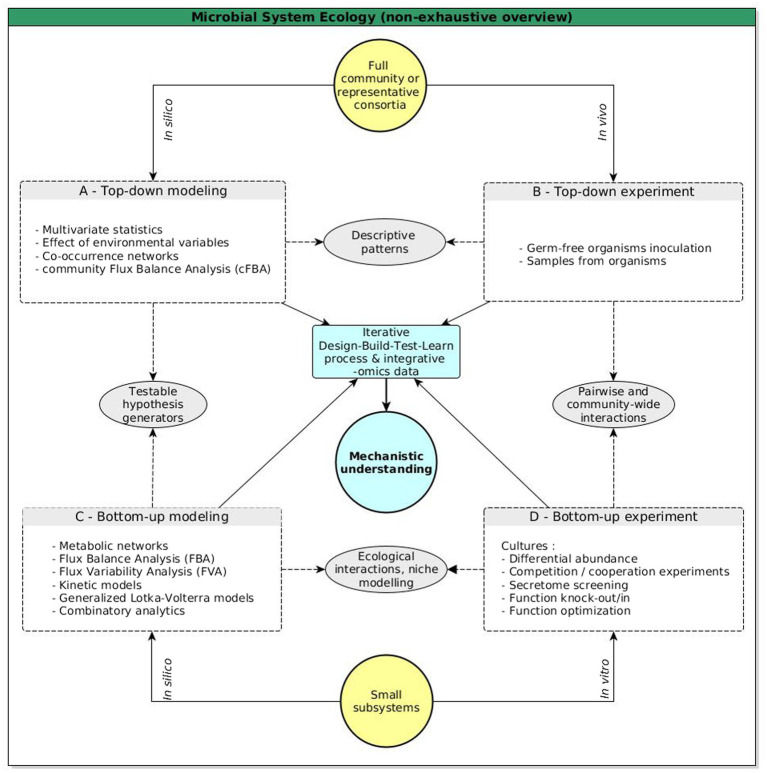
Schematic view of top-down and bottom-up approaches in MSE. The list of methods, techniques, and goals is not exhaustive. In this framework, deciphering the structure and dynamics of a microbial community implies continuous and iterative shifts between approaches, either top-down/bottom-up or *in silico*/*in vitro*/*in vivo*. Top-down modeling **(A)** intensively used omics data obtained from high-scale top-down experiments involving numerous species **(B)**. For example, top-down models can use descriptive and multivariate statistics to detect structural and time patterns in species abundances, or cluster microorganisms in functional groups. Both can subsequently be correlated with their co-occurrences and modeled with generalized Lotka-Volterra models (respectively based on relative abundances and growth rates with an interactions matrix), which are also used to model the potential influence of a microorganisms on others. In bottom-up modeling **(C)**, a reductionist approach is preferred, and small subsystems of microorganisms are analyzed in more detail, with emphasis on modeling how they putatively interact. Most models are based on reconstructed metabolic networks, which are crucial to predict interactions such as nutrient competition or exchange. Software based on constraint-based programming and answer-set programming exist to rapidly find combinations that can then be further modeled using flux analysis or regular Lotka-Volterra models. Putative interactions must be tested when possible **(D)**. Each approach and method used contributes its own knowledge and should be completed with other knowledge. Approaches must be chosen based on the research goal: microbiome engineering, synthetic biology, and deciphering assembly rules of the community with a mechanistic and holistic view (etc.). Methods and techniques are provided as examples and do not claim to be exhaustive (see Shahzad and Loor, 2012; Franzosa et al., 2015; Amor and Bello, 2019; Lawson et al., 2019; Lloyd-Price et al., 2019; Vrancken et al., 2019 for more).

Deciphering microbial interactions is a major challenge in microbiome research to enable the shift from descriptive approaches to a mechanistic understanding of microbiome assemblages. Such complex systems involving hundreds of interacting organisms make it difficult to determine which interactions primarily drive community stability or modulate shifts in assembly trajectories. In the following sections, we discuss the potential of MSE (Figures 1E, 2), which crosses the cellular and population scales with combined top-down and bottom-up approaches to disentangle the mechanisms of cooperation and co-existence in a microbiome.

## Microbial Systems Ecology: a Crossroad Between Systems Biology, Cellular Biology, and Community Ecology

Systems biology is the computational and mathematical study of interactions between the components of biological entities (molecules, cells, organs, and organisms), considered as complex systems (Snoep and Westerhoff, 2005). Connecting components is preferred over characterizing isolated parts (Kitano, 2002b), because the latter are not sufficient to understand the behavior of the system as a whole. System biology involves a cycle of theory, modeling, and testing hypotheses, followed by experimental validation. In addition to the structure of the system (gene interactions, biochemical pathways, etc.), biological systems must integrate dynamics and robustness of components, i.e., how they behave over time under varying conditions, as well as their sensitivity to perturbations (Kitano, 2002b; Alon, 2006). Omics approaches produce sufficient quantitative data to support simulation-based research, leading to genome-scale modeling to analyze the cell function properties of the system, mainly based on graph theory (Kitano, 2002a; Rodriguez et al., 2019). Research includes reconstruction of metabolic networks, transcriptional regulatory networks, interactome networks, and hormone signaling (etc.), for various applications including crop protection or sustainable agriculture, therapies for obesity, diabetes, and inflammatory bowel disease, or conservation biology (Amor and Bello, 2019; Rodriguez et al., 2019; Vázquez-Castellanos et al., 2019).

Microbial systems ecology is defined as the holistic study of microbial communities using systems biology (Muller et al., 2018). In microbiology, the cellular and the individual levels are often intertwined: the components of the system are cells and are also individuals of different microbial species and components of the community, creating a crossroad which, in MSE, is used to analyze populations and communities. MSE includes numerous approaches to study assembly rules, co-existence, and trophic networks (etc.) in microbial communities. Such communities are seen as networks of networks: i.e., community members consisting of collections of interwoven molecular networks (Muller et al., 2018). MSE is mainly based on the construction of predictive models using a corpus of computational methods that make it possible to mine large amounts of data, notably to predict putative interactions or phenotypes under different growth conditions (Franzosa et al., 2015; Bordron et al., 2016). Nevertheless, most of these methods are still vulnerable to mistakes *via* unmeasured external factors. They are therefore often treated as hypothesis generators, of which the strongest have to be tested experimentally (Coyte and Rakoff-Nahoum, 2019; Goyal et al., 2021).

Considerable efforts have been made to analyze and model microbiomes and predict microbial interactions (Li et al., 2016; Knight et al., 2018; Kumar et al., 2019)notably based on predicted metabolism by identifying keystone genes and functions and by identifying the microorganisms’ ecological niches. Genome-encoded metabolism can reveal fundamental niches while resource usage, realized niches, and their overlaps between species can be inferred from transcriptomes, proteomes, and metabolomes (Mee et al., 2014; Muller et al., 2018), thus making it possible to identify core and specific metabolism or to predict metabolic interactions. For example, in a set of five bacteria, such models found that species-specific metabolism is related to secondary metabolism, and metabolic cooperation was required to perform copper bioleaching, an important biohydrometallurgic process in ancient microbial communities that also harbors an economic interest (Bordron et al., 2016). Such a community was also chosen for its simplicity, allowing a reductionist approach while maintaining realistic ecological conditions. Despite the immense potential of omics, niche inference remains a challenging task due to niche multi-dimensionality, the complexity of trophic interactions, and fluctuating environmental conditions (Muller et al., 2018). Hence, based on systems biology and on the crossroads of cellular and community scales, MSE developed multiple frameworks, each dedicated to investigate specific aspects of microbial communities. However, in order to obtain a holistic and mechanistic view, an integration of all approaches is required.

### Microbial Systems Ecology Approaches and Framework

#### Metabolic Network Reconstruction

Once an organism’s genome has been sequenced and annotated, its metabolic network can be inferred (Mendoza et al., 2019). Metabolic networks are often referred to as “genome-scale metabolic models” (GEMs) gathering all the metabolic capacities of an organism, linking chemical reactions, reactants, products, and enzymes needed to reconstruct metabolic pathways (Jansma and El Aidy, 2020). GEMs can predict cell behavior under various conditions (notably nutritional): which metabolic functions organisms are capable of achieving, which compounds can be produced, or what are the growth requirements of a particular network. However, the main limit is that GEMs are mainly drafts, and their reliability depends to a great extent on how well annotated an organism already is. This applies to only a few dozen well-known organisms including humans, the mouse, *Arabidopsis thaliana*, some yeast, and bacteria (Shahzad and Loor, 2012). Under-investigated organisms produce more general GEMs, because specific genes are less annotated, resulting in gaps or incomplete pathways, which is problematic when attempting to establish precise functional profiles (Jansma and El Aidy, 2020). Indeed, it has been demonstrated that many GEMs are limited to well-conserved, primary metabolic pathways rather than secondary metabolic pathways, thus limiting the representation of the organisms they model (Monk et al., 2014). Such problems can be overcome with additional steps like gap-filling and manual curation (Prigent et al., 2017), but these are subject to false positives when working with unknown organisms (Henry et al., 2010; Frioux et al., 2020).

When data on stoichiometric reactions are available, metabolic networks can be enhanced through quantitative analysis of metabolite fluxes within the network. After considering available nutrients, fluxes of metabolites within and between pathways are computed to maximize an objective function, such as biomass production. A standard approach is flux balance analysis (Bordbar et al., 2014). However, the objective function is often difficult to define, and such methods require high-quality GEMs. What is more, they still only provide a static view of the community. Metabolic modeling, dynamics of species abundance, as well as concentrations of metabolites over time are an active field of development (Muller et al., 2018; Vrancken et al., 2019). For recent reviews of computational tools dedicated to the reconstruction and analysis of metabolic networks, we recommend the ones by Mendoza et al. (2019)and García-Jiménez et al. (2021).

Simulations of GEMs under environmental constraints are used to identify potential competition for nutrients and to predict cross-feeding or ecological niches, with applications in metabolic engineering (Heinken and Thiele, 2015; Magnúsdóttir et al., 2017; Frioux et al., 2018; Muller et al., 2018; Mendoza et al., 2019). These approaches enable more direct quantification of interactions than techniques that rely on natural communities *in vivo*. However, problems increase with the number of species studied simultaneously, and precise metabolic modeling rapidly becomes impractical for natural communities because of the tremendous number of possible configurations (Coyte and Rakoff-Nahoum, 2019). Specific approaches consider multiple species at once, for example, community flux balance analysis (Khandelwal et al., 2013). Nonetheless, methodological limits do not produce the necessary holistic understanding of microbiota (Vandenkoornhuyse et al., 2010). They rather give an only slightly more than general overview of emergent properties, or are limited to a small fraction of a community. In summary, metabolic networks can model an organism’s functioning and are thus mostly used in bottom-up (reductionist) approaches (Shahzad and Loor, 2012), but it is important to also take top-down (global) approaches or combinations of both into consideration (Figure 2; Lawson et al., 2019).

#### The Microbial Systems Ecology Framework Calls for Shifts Between Top-Down and Bottom-Up Approaches

In MSE, the study of complex systems like microbiomes uses both top-down and bottom-up approaches within a design-build-test-learn process that is particularly suitable for microbiome engineering and synthetic biology (Figure 2), where the optimum and minimum combinations of organisms are investigated in order to perform a biological function (for an exhaustive explanation and review, see Lawson et al., 2019). Such a process works in cycles, where the design and build phases are adapted to the functions targeted, and the test and learn phases are used to correct any errors and to optimize the system.

Top-down approaches start from a complete microbial community (or at least a sufficiently big and representative set of microorganisms) and aim to discover signature patterns of underlying biological mechanisms (Figures 2A,B). Top-down approaches are basically descriptive and were developed using many multivariate statistics, meta-omics, and experimental data, to capture key microbiome functions or effects or particular environmental variables rather than prioritizing which organism or pathway is at play behind an observed phenotype (Ramette, 2007; Shahzad and Loor, 2012; Lawson et al., 2019). Most of our knowledge about the gut microbiome was obtained using top-down approaches and helped discern dysbiosis patterns associated with diseases (Bashan et al., 2016; Amor and Bello, 2019). For example, one method involves clustering the members of a community according to their metabolic functions, and/or building co-occurrence networks to identify coexistences and to propose hypotheses to explain the origin of the coexistence (Faust and Raes, 2012; Layeghifard et al., 2017). To achieve that goal, simple metrics computed from metabolic networks are used to compute metabolic overlap, metabolic interaction potential, or the functional distance between organisms (Zelezniak et al., 2015; Russel et al., 2017). Such metrics allow the formulation of hypotheses about ecological processes involved, including metabolic interactions. Top-down approaches offer a macro-scale framework to decipher overall functions of a microbial community, as well as its resistance and resilience. However these approaches overlook intricate details, notably regarding the multiple ecological interactions between microorganisms that lead to the emergence of the observed functions. Top-down approaches are consequently limited in terms of getting holistic and mechanistic views of complex (i.e., natural) communities (Vrancken et al., 2019).

This limit is offset by bottom-up approaches that ignore the whole system and start from single microorganisms to build simple sub-communities to deduce the functional properties that could emerge from a small subsystem, and then gradually increase model complexity (Figures 2C,D, Amor and Bello, 2019; Lawson et al., 2019). Bottom-up approaches use proficient computational and mathematical modeling (for details, see Vrancken et al., 2019), notably based on GEMs, for example with constraint-based analytics able to directly identify combinations of GEMs able to produce a compound that cannot be produced by single genomes, such as in Frioux et al. (2018). Using an Answer Set Programming (ASP) method, an exhaustive screening involving 2,051 bacterial GEMs from the Human Microbiome Project was carried out. It allowed to compute tremendous possible combinations of bacteria able to perform a function through cross-feeding. Such approaches have (for example) been used to predict mutualism and competition in relatively big microbial consortia (Friedman et al., 2017; Kong et al., 2018). Species co-existence can also be mathematically modeled, for instance with the use of generalized Lotka-Voltera models. Such models compute the growth rate of any species in a community, while taking into account its interactions (known or hypothesized, then parameterized by the user) with all the other microorganisms (Coyte and Rakoff-Nahoum, 2019). Overall, cooperative interactions (including metabolic dependencies) are often key components of bottom-up designs in synthetic biology (Amor and Bello, 2019)and in general, core metabolism is a reliable starting point, as it captures carbon and energy metabolism (Lawson et al., 2019).

To sum it up, top-down and bottom-up approaches start at opposite ends, depending on the researched patterns. They complement each other in order to progress iteratively toward a mechanistic view of a complete microbial community.

## Conclusion

Deciphering ecological processes taking place within a microbial community is the only way to obtain a mechanistic view of its functioning. Ecological interactions, particularly cross-feeding, must thus be taken into account in any microbial ecology project, notably in synthetic biology and microbiome engineering, with many applications including human health and sustainable agriculture (Toju et al., 2018; Henriques et al., 2020). With this goal in view, MSE frameworks are being developed to unify top-down and bottom-up approaches in an iterative design-build-test-learn cycle (Lawson et al., 2019). Still, MSE should be used cautiously to avoid being drowned under hundreds of irrelevant models. Whenever possible, predictions of an MSE framework should be tested experimentally (Röling and Van Bodegom, 2014; Muller et al., 2018; Vázquez-Castellanos et al., 2019), and in return, experimental observations should improve models. To build reliable and in-depth knowledge, efforts should focus on a few aspects, such as GEM quality (in order to go beyond research on conserved, well-known metabolic pathways), the integration of -omics data (Franzosa et al., 2015), notably the microbial secretome with exometabolomics, and cross-talk with other approaches such as niche modeling or dynamics modeling (Jacoby and Kopriva, 2019).

## Author Contributions

AM is the first author, who did the bibliography and wrote the text and figures. PV, NV, and SH contributed equally to make corrections and suggestions. All authors contributed to the article and approved the submitted version.

## Funding

This work was supported by a grant from the French Ministry for Research and Innovation, by a grant from the CNRS (EC2CO), and also by a starting grant to SH from a European Research Council (MICRORULES 758003), the Max Planck Institute, the Cluster of Excellence on Plant Sciences (CEPLAS), and the “Priority Program: Deconstruction and Reconstruction of the Plant Microbiota (SPP DECRyPT 2125),” both funded by the Deutsche Forschungsgemeinschaft.

## Conflict of Interest

The authors declare that the research was conducted in the absence of any commercial or financial relationships that could be construed as a potential conflict of interest.

## Publisher’s Note

All claims expressed in this article are solely those of the authors and do not necessarily represent those of their affiliated organizations, or those of the publisher, the editors and the reviewers. Any product that may be evaluated in this article, or claim that may be made by its manufacturer, is not guaranteed or endorsed by the publisher.

## Glossary

**Metabolite cross-feeding or by-product cross-feeding (unidirectional) :** The compounds on which the receiver feeds are molecules that cannot be further metabolized by the producer. They are waste products, potentially toxic, thus detoxified by beneficial microorganisms (Smith et al., 2019). Hence, the exchange results from an initial selfish behavior of the producer, which gets rid of its waste or generates many metabolites by metabolic overflow due to its capacity to better, compete for initial nutrients (Carlson et al., 2018; D’Souza et al., 2018).

**Substrate cross-feeding (unidirectional) :** The secreted compounds may be further metabolized by both organisms. For example, extracellular enzymes responsible for degrading complex molecules, whose products will subsequently be assimilated (Smith et al., 2019).

**Mutual cross-feeding (bidirectional) :** The case in which both microorganisms feed on metabolites produced and secreted by the other. In this case, the difference must be made between opportunistic and truly cooperative behaviors. Augmented cross-feeding (also referred to as cooperative cross-feeding or by-product reciprocity) describes true cooperation and is based on the energetic investment that increases the quantity of the cross-fed compounds (D’Souza et al., 2018; Smith et al., 2019).

**Black Queen Hypothesis :** Evolutionary trajectory by gene loss(es) of beneficiaries dependent on providers of public goods have been formalized in the Black Queen Hypothesis (BQH, Morris et al., 2012). This hypothesis relies on the emergence of the capacity loss to produce a costly common good within a community or a population leading to a steady state among providers and beneficiaries of the common good (Mas et al., 2016). The BQH of evolution also explains an evolutionary trajectory of competition avoidance toward a steady-state equilibrium for the coexistence of microorganisms (Mas et al., 2016).

**Evolutionary game theory :** Game theory aims to determine the optimal strategies to adopt in competitive games. Evolutionary game theory applied its corpus to evolving populations in biology, the strategies being (roughly) how species adapt their behavior to maintain or increase the fitness of their population. Strategies were extended to cooperative and altruistic behaviors that were at some point thought incompatible with Darwinian evolution. Evolutionary game theory brought new insight on the existence of cooperation by applying natural selection to the group level rather than to the individual level, i.e., the benefit of the species rather than the benefit of the individual (Maynard Smith and Price, 1973; Maynard Smith, 1982; Mirowski, 1992; Cohen, 2004).
